# Optimal database combinations for literature searches in systematic reviews: a prospective exploratory study

**DOI:** 10.1186/s13643-017-0644-y

**Published:** 2017-12-06

**Authors:** Wichor M. Bramer, Melissa L. Rethlefsen, Jos Kleijnen, Oscar H. Franco

**Affiliations:** 1 Medical Library, Erasmus MC, Erasmus University Medical Centre Rotterdam, 3000 CS Rotterdam, the Netherlands; 2 Spencer S. Eccles Health Sciences Library, University of Utah, Salt Lake City, Utah USA; 3 Kleijnen Systematic Reviews Ltd., York, UK; 4 School for Public Health and Primary Care (CAPHRI), Maastricht University, Maastricht, the Netherlands; 5 Department of Epidemiology, Erasmus MC, Erasmus University Medical Centre Rotterdam, Rotterdam, the Netherlands

**Keywords:** Databases, bibliographic, Review literature as topic, Sensitivity and specificity, Information storage and retrieval

## Abstract

**Background:**

Within systematic reviews, when searching for relevant references, it is advisable to use multiple databases. However, searching databases is laborious and time-consuming, as syntax of search strategies are database specific. We aimed to determine the optimal combination of databases needed to conduct efficient searches in systematic reviews and whether the current practice in published reviews is appropriate. While previous studies determined the coverage of databases, we analyzed the actual retrieval from the original searches for systematic reviews.

**Methods:**

Since May 2013, the first author prospectively recorded results from systematic review searches that he performed at his institution. PubMed was used to identify systematic reviews published using our search strategy results. For each published systematic review, we extracted the references of the included studies. Using the prospectively recorded results and the studies included in the publications, we calculated recall, precision, and number needed to read for single databases and databases in combination. We assessed the frequency at which databases and combinations would achieve varying levels of recall (i.e., 95%). For a sample of 200 recently published systematic reviews, we calculated how many had used enough databases to ensure 95% recall.

**Results:**

A total of 58 published systematic reviews were included, totaling 1746 relevant references identified by our database searches, while 84 included references had been retrieved by other search methods. Sixteen percent of the included references (291 articles) were only found in a single database; Embase produced the most unique references (*n* = 132). The combination of Embase, MEDLINE, Web of Science Core Collection, and Google Scholar performed best, achieving an overall recall of 98.3 and 100% recall in 72% of systematic reviews. We estimate that 60% of published systematic reviews do not retrieve 95% of all available relevant references as many fail to search important databases. Other specialized databases, such as CINAHL or PsycINFO, add unique references to some reviews where the topic of the review is related to the focus of the database.

**Conclusions:**

Optimal searches in systematic reviews should search at least Embase, MEDLINE, Web of Science, and Google Scholar as a minimum requirement to guarantee adequate and efficient coverage.

**Electronic supplementary material:**

The online version of this article (10.1186/s13643-017-0644-y) contains supplementary material, which is available to authorized users.

## Background

Investigators and information specialists searching for relevant references for a systematic review (SR) are generally advised to search multiple databases and to use additional methods to be able to adequately identify all literature related to the topic of interest [1–6]. The Cochrane Handbook, for example, recommends the use of at least MEDLINE and Cochrane Central and, when available, Embase for identifying reports of randomized controlled trials [7]. There are disadvantages to using multiple databases. It is laborious for searchers to translate a search strategy into multiple interfaces and search syntaxes, as field codes and proximity operators differ between interfaces. Differences in thesaurus terms between databases add another significant burden for translation. Furthermore, it is time-consuming for reviewers who have to screen more, and likely irrelevant, titles and abstracts. Lastly, access to databases is often limited and only available on subscription basis.

Previous studies have investigated the added value of different databases on different topics [8–15]. Some concluded that searching only one database can be sufficient as searching other databases has no effect on the outcome [16, 17]. Nevertheless others have concluded that a single database is not sufficient to retrieve all references for systematic reviews [18, 19]. Most articles on this topic draw their conclusions based on the coverage of databases [14]. A recent paper tried to find an acceptable number needed to read for adding an additional database; sadly, however, no true conclusion could be drawn [20]. However, whether an article is present in a database may not translate to being found by a search in that database. Because of this major limitation, the question of which databases are necessary to retrieve all relevant references for a systematic review remains unanswered. Therefore, we research the probability that single or various combinations of databases retrieve the most relevant references in a systematic review by studying actual retrieval in various databases.

The aim of our research is to determine the combination of databases needed for systematic review searches to provide efficient results (i.e., to minimize the burden for the investigators without reducing the validity of the research by missing relevant references). A secondary aim is to investigate the current practice of databases searched for published reviews. Are included references being missed because the review authors failed to search a certain database?

## Methods

### Development of search strategies

At Erasmus MC, search strategies for systematic reviews are often designed via a librarian-mediated search service. The information specialists of Erasmus MC developed an efficient method that helps them perform searches in many databases in a much shorter time than other methods. This method of literature searching and a pragmatic evaluation thereof are published in separate journal articles [21, 22]. In short, the method consists of an efficient way to combine thesaurus terms and title/abstract terms into a single line search strategy. This search is then optimized. Articles that are indexed with a set of identified thesaurus terms, but do not contain the current search terms in title or abstract, are screened to discover potential new terms. New candidate terms are added to the basic search and evaluated. Once optimal recall is achieved, macros are used to translate the search syntaxes between databases, though manual adaptation of the thesaurus terms is still necessary.

Review projects at Erasmus MC cover a wide range of medical topics, from therapeutic effectiveness and diagnostic accuracy to ethics and public health. In general, searches are developed in MEDLINE in Ovid (Ovid MEDLINE® In-Process & Other Non-Indexed Citations, Ovid MEDLINE® Daily and Ovid MEDLINE®, from 1946); Embase.com (searching both Embase and MEDLINE records, with full coverage including Embase Classic); the Cochrane Central Register of Controlled Trials (CENTRAL) via the Wiley Interface; Web of Science Core Collection (hereafter called Web of Science); PubMed restricting to records in the subset “as supplied by publisher” to find references that not yet indexed in MEDLINE (using the syntax publisher [sb]); and Google Scholar. In general, we use the first 200 references as sorted in the relevance ranking of Google Scholar. When the number of references from other databases was low, we expected the total number of potential relevant references to be low. In this case, the number of hits from Google Scholar was limited to 100. When the overall number of hits was low, we additionally searched Scopus, and when appropriate for the topic, we included CINAHL (EBSCOhost), PsycINFO (Ovid), and SportDiscus (EBSCOhost) in our search.

Beginning in May 2013, the number of records retrieved from each search for each database was recorded at the moment of searching. The complete results from all databases used for each of the systematic reviews were imported into a unique EndNote library upon search completion and saved without deduplication for this research. The researchers that requested the search received a deduplicated EndNote file from which they selected the references relevant for inclusion in their systematic review. All searches in this study were developed and executed by W.M.B.

### Determining relevant references of published reviews

We searched PubMed in July 2016 for all reviews published since 2014 where first authors were affiliated to Erasmus MC, Rotterdam, the Netherlands, and matched those with search registrations performed by the medical library of Erasmus MC. This search was used in earlier research [21]. Published reviews were included if the search strategies and results had been documented at the time of the last update and if, at minimum, the databases Embase, MEDLINE, Cochrane CENTRAL, Web of Science, and Google Scholar had been used in the review. From the published journal article, we extracted the list of final included references. We documented the department of the first author. To categorize the types of patient/population and intervention, we identified broad MeSH terms relating to the most important disease and intervention discussed in the article. We copied from the MeSH tree the top MeSH term directly below the disease category or, in to case of the intervention, directly below the therapeutics MeSH term. We selected the domain from a pre-defined set of broad domains, including therapy, etiology, epidemiology, diagnosis, management, and prognosis. Lastly, we checked whether the reviews described limiting their included references to a particular study design.

To identify whether our searches had found the included references, and if so, from which database(s) that citation was retrieved, each included reference was located in the original corresponding EndNote library using the first author name combined with the publication year as a search term for each specific relevant publication. If this resulted in extraneous results, the search was subsequently limited using a distinct part of the title or a second author name. Based on the record numbers of the search results in EndNote, we determined from which database these references came. If an included reference was not found in the EndNote file, we presumed the authors used an alternative method of identifying the reference (e.g., examining cited references, contacting prominent authors, or searching gray literature), and we did not include it in our analysis.

### Data analysis

We determined the databases that contributed most to the reviews by the number of unique references retrieved by each database used in the reviews. Unique references were included articles that had been found by only one database search. Those databases that contributed the most unique included references were then considered candidate databases to determine the most optimal combination of databases in the further analyses.

In Excel, we calculated the performance of each individual database and various combinations. Performance was measured using recall, precision, and number needed to read. See Table 1 for definitions of these measures. These values were calculated both for all reviews combined and per individual review.

**Table 1:** Definitions of general measures of performance in searches

Recall	$\frac{\#\mathrm{included}\ \mathrm{references}\ \mathrm{retrieved}\ \mathrm{by}\ \mathrm{a}\ \mathrm{database}/\mathrm{combination}}{\#\mathrm{included}\ \mathrm{references}\ \mathrm{retrieved}\ \mathrm{by}\ \mathrm{a}\mathrm{ll}\ \mathrm{database}\mathrm{s}}$
Precision	$\frac{\#\mathrm{included}\ \mathrm{references}\ \mathrm{retrieved}\ \mathrm{by}\ \mathrm{a}\ \mathrm{database}/\mathrm{combination}}{\#\mathrm{total}\ \mathrm{references}\ \mathrm{retrieved}\ \mathrm{by}\ \mathrm{those}\ \mathrm{database}\left(\mathrm{s}\right)}$
Number Needed to Read	$\frac{\#\mathrm{total}\ \mathrm{references}\ \mathrm{retrieved}\ \mathrm{by}\ \mathrm{a}\ \mathrm{database}/\mathrm{combination}}{\#\mathrm{included}\ \mathrm{references}\ \mathrm{retrieved}\ \mathrm{by}\ \mathrm{those}\ \mathrm{database}\left(\mathrm{s}\right)}$

Performance of a search can be expressed in different ways. Depending on the goal of the search, different measures may be optimized. In the case of a clinical question, precision is most important, as a practicing clinician does not have a lot of time to read through many articles in a clinical setting. When searching for a systematic review, recall is the most important aspect, as the researcher does not want to miss any relevant references. As our research is performed on systematic reviews, the main performance measure is recall.

We identified all included references that were uniquely identified by a single database. For the databases that retrieved the most unique included references, we calculated the number of references retrieved (after deduplication) and the number of included references that had been retrieved by all possible combinations of these databases, in total and per review. For all individual reviews, we determined the median recall, the minimum recall, and the percentage of reviews for which each single database or combination retrieved 100% recall.

For each review that we investigated, we determined what the recall was for all possible different database combinations of the most important databases. Based on these, we determined the percentage of reviews where that database combination had achieved 100% recall, more than 95%, more than 90%, and more than 80%. Based on the number of results per database both before and after deduplication as recorded at the time of searching, we calculated the ratio between the total number of results and the number of results for each database and combination.

Improvement of precision was calculated as the ratio between the original precision from the searches in all databases and the precision for each database and combination.

To compare our practice of database usage in systematic reviews against current practice as evidenced in the literature, we analyzed a set of 200 recent systematic reviews from PubMed. On 5 January 2017, we searched PubMed for articles with the phrase “systematic review” in the title. Starting with the most recent articles, we determined the databases searched either from the abstract or from the full text until we had data for 200 reviews. For the individual databases and combinations that were used in those reviews, we multiplied the frequency of occurrence in that set of 200 with the probability that the database or combination would lead to an acceptable recall (which we defined at 95%) that we had measured in our own data.

## Results

Our earlier research had resulted in 206 systematic reviews published between 2014 and July 2016, in which the first author was affiliated with Erasmus MC [21]. In 73 of these, the searches and results had been documented by the first author of this article at the time of the last search. Of those, 15 could not be included in this research, since they had not searched all databases we investigated here. Therefore, for this research, a total of 58 systematic reviews were analyzed. The references to these reviews can be found in Additional file 1. An overview of the broad topical categories covered in these reviews is given in Table 2. Many of the reviews were initiated by members of the departments of surgery and epidemiology. The reviews covered a wide variety of disease, none of which was present in more than 12% of the reviews. The interventions were mostly from the chemicals and drugs category, or surgical procedures. Over a third of the reviews were therapeutic, while slightly under a quarter answered an etiological question. Most reviews did not limit to certain study designs, 9% limited to RCTs only, and another 9% limited to other study types.

**Table 2:** Description of topics of included references (only values above 5% are shown)

Department (*N* = 55)	
Surgery	13 (24%)
Epidemiology	10 (18%)
Internal medicine	3 (5%)
Orthopedics	3 (5%)
Patient (*N* = 52)	
Neoplasms	6 (12%)
Wounds and injuries	6 (12%)
Musculoskeletal diseases	5 (10%)
Cardiovascular diseases	5 (10%)
Nutritional and metabolic diseases	5 (10%)
Intervention (*N* = 31)	
Chemicals and drugs category	12 (39%)
Surgical procedures, operative	8 (26%)
Food and beverages	2 (6%)
Biological factors	2 (6%)
Domain (*N* = 54)	
Therapy	19 (35%)
Etiology	13 (24%)
Epidemiology	6 (11%)
Diagnosis	6 (11%)
Management	5 (9%)
Prognosis	5 (9%)
Study types (*N* = 58)	
No limits mentioned	48 (83%)
RCTs	5 (9%)
RCTs and cohort studies/case control studies	5 (9%)

Together, these reviews included a total of 1830 references. Of these, 84 references (4.6%) had not been retrieved by our database searches and were not included in our analysis, leaving in total 1746 references. In our analyses, we combined the results from MEDLINE in Ovid and PubMed (the subset as supplied by publisher) into one database labeled MEDLINE.

### Unique references per database

A total of 292 (17%) references were found by only one database. Table 3 displays the number of unique results retrieved for each single database. Embase retrieved the most unique included references, followed by MEDLINE, Web of Science, and Google Scholar. Cochrane CENTRAL is absent from the table, as for the five reviews limited to randomized trials, it did not add any unique included references. Subject-specific databases such as CINAHL, PsycINFO, and SportDiscus only retrieved additional included references when the topic of the review was directly related to their special content, respectively nursing, psychiatry, and sports medicine.

**Table 3:** Number of unique included references by each specific database

Database	Number of reviews that used the database	Number of reviews with unique references	Number of unique references
Embase	58	29 (50%)	132 (45%)
MEDLINE	58	27 (47%)	69 (24%)
Web of Science	58	19 (33%)	37 (13%)
Google Scholar	58	24 (41%)	37 (13%)
CINAHL	18	1 (6%)	6 (2%)
Scopus	24	3 (13%)	5 (2%)
PsycINFO	11	1 (9%)	2 (1%)
SportDiscus	2	2 (100%)	3 (1%)

### Overall performance

The four databases that had retrieved the most unique references (Embase, MEDLINE, Web of Science, and Google Scholar) were investigated individually and in all possible combinations (see Table 4). Of the individual databases, Embase had the highest overall recall (85.9%). Of the combinations of two databases, Embase and MEDLINE had the best results (92.8%). Embase and MEDLINE combined with either Google Scholar or Web of Science scored similarly well on overall recall (95.9%). However, the combination with Google Scholar had a higher precision and higher median recall, a higher minimum recall, and a higher proportion of reviews that retrieved all included references. Using both Web of Science and Google Scholar in addition to MEDLINE and Embase increased the overall recall to 98.3%. The higher recall from adding extra databases came at a cost in number needed to read (NNR). Searching only Embase produced an NNR of 57 on average, whereas, for the optimal combination of four databases, the NNR was 73.

**Table 4:** Performance of several databases and database combinations in terms of sensitivity and precision

	# results	# includes (*N* = 1746)	Overall recall^a^	Median recall^b^	Minimum recall^c^	Percentage 100% recall^d^	Precision^e^	Number needed to read^f^
Embase (EM)	85,521	1500	85.9%	87.3%	45.8%	13.8%	1.8%	57
MEDLINE (ML)	56,340	1375	78.8%	82.9%	50.0%	8.6%	2.4%	41
Web of Science (WoS)	48,561	1189	68.1%	72.5%	13.2%	6.9%	2.4%	41
Google Scholar (GS)	10,342	601	34.4%	38.0%	8.3%	5.2%	5.8%	17
EM-ML	100,444	1621	92.8%	94.6%	66.7%	24.1%	1.6%	62
EM-WoS	104,444	1585	90.8%	93.8%	57.9%	27.6%	1.5%	66
EM-GS	91,411	1570	89.9%	93.3%	65.8%	25.9%	1.7%	58
ML-WoS	75,263	1481	84.8%	87.1%	60.0%	15.5%	2.0%	51
ML-GS	62,230	1459	83.6%	89.8%	63.2%	15.5%	2.3%	43
WoS-GS	54,451	1320	75.6%	85.7%	23.7%	13.8%	2.4%	41
EM-ML-GS	106,334	1674	95.9%	97.4%	78.9%	41.4%	1.6%	64
EM-ML-WoS	119,367	1674	95.9%	97.1%	71.1%	37.9%	1.4%	70
EM-WoS-GS	110,334	1638	93.8%	98.1%	65.8%	44.8%	1.5%	67
ML-WoS-GS	81,153	1528	87.5%	92.6%	70.0%	29.3%	1.9%	53
EM-ML-GS-WoS	125,257	1716	98.3%	100.0%	78.9%	74.1%	1.4%	73

^a^Overall recall: The total number of included references retrieved by the databases divided by the total number of included references retrieved by all databases

^b^Median recall: The median value of recall per review

^c^Minimum recall: The lowest value of recall per review

^d^Percentage 100% recall: The percentage of reviews for which the database combination retrieved all included references

^e^Precision: The number of included references divided by the total number of references retrieved

^f^Number Needed to Read: The total number of references retrieved divided by the number of included references

### Probability of appropriate recall

**Fig. 1:**
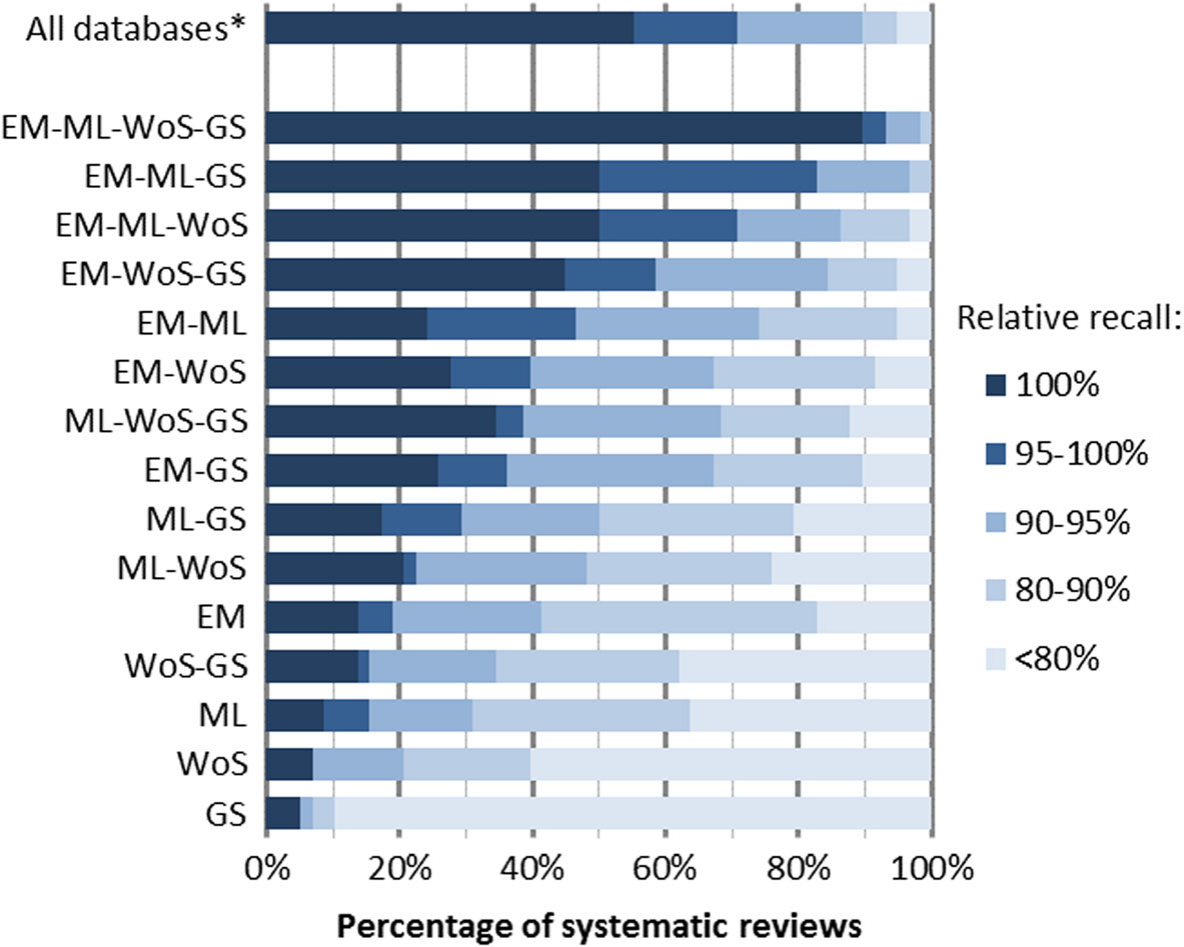
Percentage of systematic reviews for which a certain database combination reached a certain recall. The *X*-axis represents the percentage of reviews for which a specific combination of databases, as shown on the *y*-axis, reached a certain recall (represented with bar colors). Abbreviations: EM Embase, ML MEDLINE, WoS Web of Science, GS Google Scholar. Asterisk indicates that the recall of all databases has been calculated over all included references. The recall of the database combinations was calculated over all included references retrieved by any database

We calculated the recall for individual databases and databases in all possible combination for all reviews included in the research. Figure 1 shows the percentages of reviews where a certain database combination led to a certain recall. For example, in 48% of all systematic reviews, the combination of Embase and MEDLINE (with or without Cochrane CENTRAL; Cochrane CENTRAL did not add unique relevant references) reaches a recall of at least 95%. In 72% of studied systematic reviews, the combination of Embase, MEDLINE, Web of Science, and Google Scholar retrieved all included references. In the top bar, we present the results of the complete database searches relative to the total number of included references. This shows that many database searches missed relevant references.

### Differences between domains of reviews

**Fig. 2:**
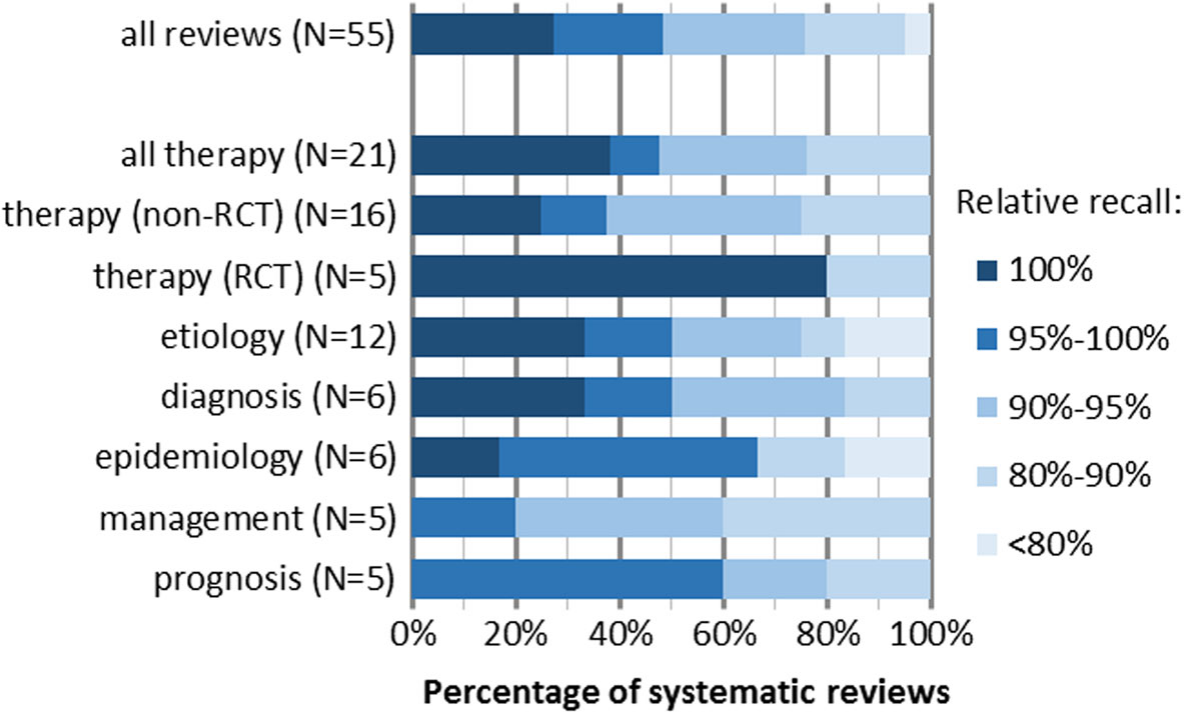
Percentage of systematic reviews of a certain domain for which the combination Embase, MEDLINE and Cochrane CENTRAL reached a certain recall

We analyzed whether the added value of Web of Science and Google Scholar was dependent of the domain of the review. For 55 reviews, we determined the domain. See Fig. 2 for the comparison of the recall of Embase, MEDLINE, and Cochrane CENTRAL per review for all identified domains. For all but one domain, the traditional combination of Embase, MEDLINE, and Cochrane CENTRAL did not retrieve enough included references. For four out of five systematic reviews that limited to randomized controlled trials (RCTs) only, the traditional combination retrieved 100% of all included references. However, for one review of this domain, the recall was 82%. Of the 11 references included in this review, one was found only in Google Scholar and one only in Web of Science.

### Reduction in number of results

**Fig. 3:**
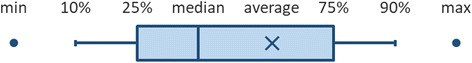
Legend of Figs. 3 and 4

**Fig. 4:**
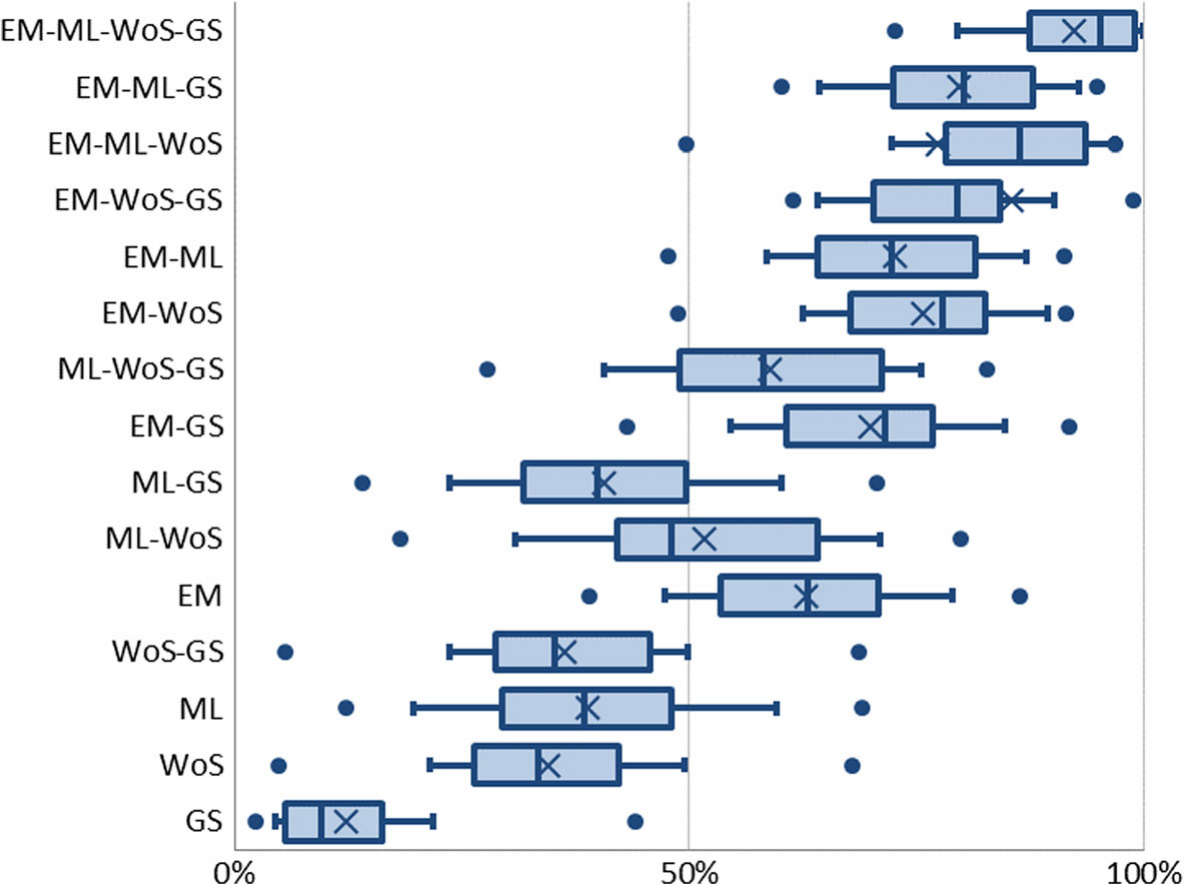
The ratio between number of results per database combination and the total number of results for all databases

**Fig. 5:**
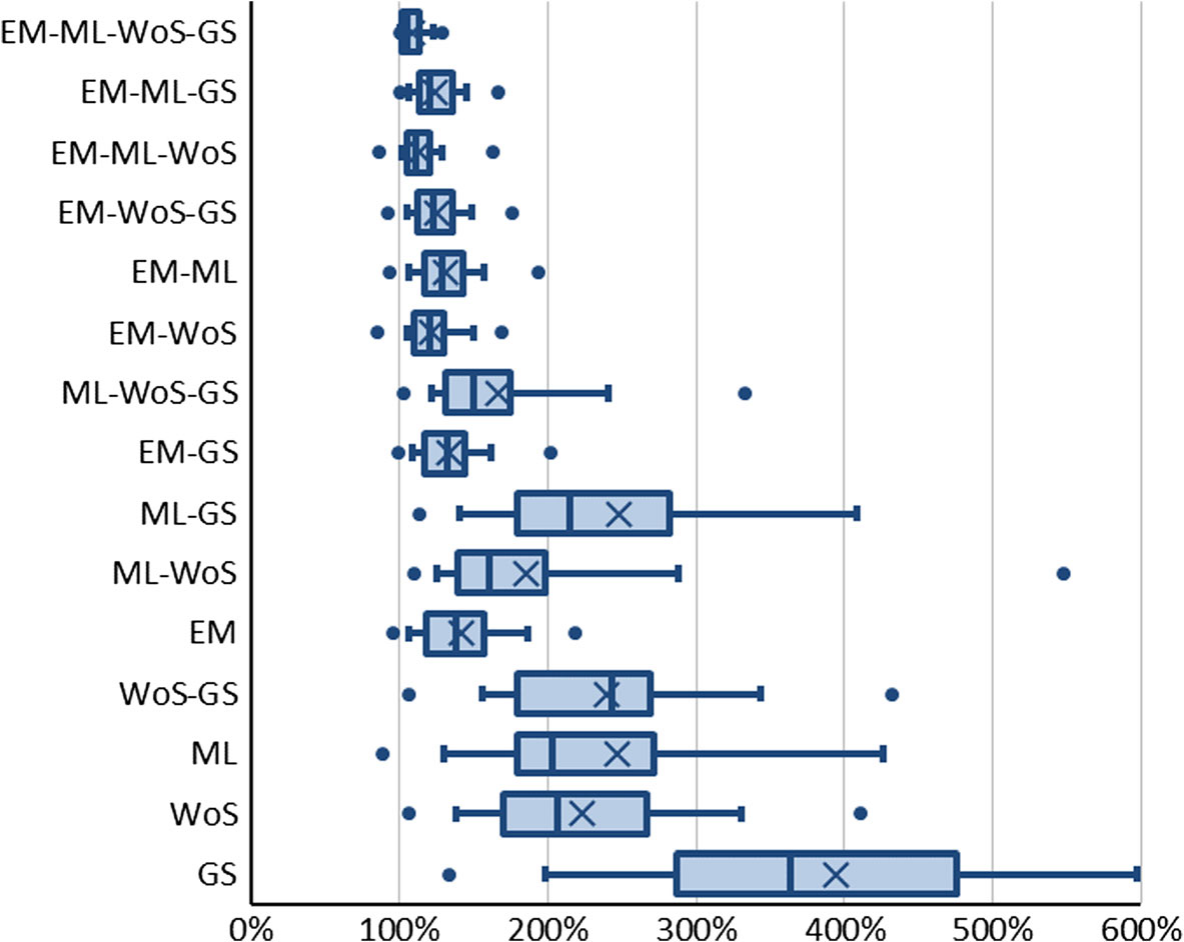
The ratio between precision per database combination and the total precision for all databases

We calculated the ratio between the number of results found when searching all databases, including databases not included in our analyses, such as Scopus, PsycINFO, and CINAHL, and the number of results found searching a selection of databases. See Fig. 3 for the legend of the plots in Figs. 4 and 5. Figure 4 shows the distribution of this value for individual reviews. The database combinations with the highest recall did not reduce the total number of results by large margins. Moreover, in combinations where the number of results was greatly reduced, the recall of included references was lower.

### Improvement of precision

To determine how searching multiple databases affected precision, we calculated for each combination the ratio between the original precision, observed when all databases were searched, and the precision calculated for different database combinations. Figure 5 shows the improvement of precision for 15 databases and database combinations. Because precision is defined as the number of relevant references divided by the number of total results, we see a strong correlation with the total number of results.

### Status of current practice of database selection

From a set of 200 recent SRs identified via PubMed, we analyzed the databases that had been searched. Almost all reviews (97%) reported a search in MEDLINE. Other databases that we identified as essential for good recall were searched much less frequently; Embase was searched in 61% and Web of Science in 35%, and Google Scholar was only used in 10% of all reviews. For all individual databases or combinations of the four important databases from our research (MEDLINE, Embase, Web of Science, and Google Scholar), we multiplied the frequency of occurrence of that combination in the random set, with the probability we found in our research that this combination would lead to an acceptable recall of 95%. The calculation is shown in Table 5. For example, around a third of the reviews (37%) relied on the combination of MEDLINE and Embase. Based on our findings, this combination achieves acceptable recall about half the time (47%). This implies that 17% of the reviews in the PubMed sample would have achieved an acceptable recall of 95%. The sum of all these values is the total probability of acceptable recall in the random sample. Based on these calculations, we estimate that the probability that this random set of reviews retrieved more than 95% of all possible included references was 40%. Using similar calculations, also shown in Table 5, we estimated the probability that 100% of relevant references were retrieved is 23%.

**Table 5:** Calculation of probability of acceptable recall of a PubMed sample of systematic reviews

	Frequency	Frequency percentage (*a*) (%)	Probability recall > 95% (*b*) (%)	*a* × *b* (%)	Probability recall 100% (*c*) (%)	*c* × *b* (%)
EM-ML	73	37	47	17	24	9
ML	41	21	16	3	9	2
EM-ML-WoS	40	20	64	13	36	7
ML-WoS	21	11	21	2	16	2
ML-GS	7	4	26	1	16	1
ML-WoS-GS	7	4	37	1	29	1
EM-ML-GS	5	3	76	2	41	1
EM	2	1	19	0	14	0
EM-WoS	1	1	40	0	28	0
WoS	1	1	7	0	7	0
Total	198^a^	100		40		23

^a^Two reviews did not use any of the databases used in this evaluation

## Discussion

Our study shows that, to reach maximum recall, searches in systematic reviews ought to include a combination of databases. To ensure adequate performance in searches (i.e., recall, precision, and number needed to read), we find that literature searches for a systematic review should, at minimum, be performed in the combination of the following four databases: Embase, MEDLINE (including Epub ahead of print), Web of Science Core Collection, and Google Scholar. Using that combination, 93% of the systematic reviews in our study obtained levels of recall that could be considered acceptable (> 95%). Unique results from specialized databases that closely match systematic review topics, such as PsycINFO for reviews in the fields of behavioral sciences and mental health or CINAHL for reviews on the topics of nursing or allied health, indicate that specialized databases should be used additionally when appropriate.

We find that Embase is critical for acceptable recall in a review and should always be searched for medically oriented systematic reviews. However, Embase is only accessible via a paid subscription, which generally makes it challenging for review teams not affiliated with academic medical centers to access. The highest scoring database combination without Embase is a combination of MEDLINE, Web of Science, and Google Scholar, but that reaches satisfactory recall for only 39% of all investigated systematic reviews, while still requiring a paid subscription to Web of Science. Of the five reviews that included only RCTs, four reached 100% recall if MEDLINE, Web of Science, and Google Scholar combined were complemented with Cochrane CENTRAL.

The Cochrane Handbook recommends searching MEDLINE, Cochrane CENTRAL, and Embase for systematic reviews of RCTs. For reviews in our study that included RCTs only, indeed, this recommendation was sufficient for four (80%) of the reviews. The one review where it was insufficient was about alternative medicine, specifically meditation and relaxation therapy, where one of the missed studies was published in the *Indian Journal of Positive Psychology*. The other study from the *Journal of Advanced Nursing* is indexed in MEDLINE and Embase but was only retrieved because of the addition of KeyWords Plus in Web of Science. We estimate more than 50% of reviews that include more study types than RCTs would miss more than 5% of included references if only traditional combination of MEDLINE, Embase, and Cochrane CENTAL is searched.

We are aware that the Cochrane Handbook [7] recommends more than only these databases, but further recommendations focus on regional and specialized databases. Though we occasionally used the regional databases LILACS and SciELO in our reviews, they did not provide unique references in our study. Subject-specific databases like PsycINFO only added unique references to a small percentage of systematic reviews when they had been used for the search. The third key database we identified in this research, Web of Science, is only mentioned as a citation index in the Cochrane Handbook, not as a bibliographic database. To our surprise, Cochrane CENTRAL did not identify any unique included studies that had not been retrieved by the other databases, not even for the five reviews focusing entirely on RCTs. If Erasmus MC authors had conducted more reviews that included only RCTs, Cochrane CENTRAL might have added more unique references.

MEDLINE did find unique references that had not been found in Embase, although our searches in Embase included all MEDLINE records. It is likely caused by difference in thesaurus terms that were added, but further analysis would be required to determine reasons for not finding the MEDLINE records in Embase. Although Embase covers MEDLINE, it apparently does not index every article from MEDLINE. Thirty-seven references were found in MEDLINE (Ovid) but were not available in Embase.com. These are mostly unique PubMed references, which are not assigned MeSH terms, and are often freely available via PubMed Central.

Google Scholar adds relevant articles not found in the other databases, possibly because it indexes the full text of all articles. It therefore finds articles in which the topic of research is not mentioned in title, abstract, or thesaurus terms, but where the concepts are only discussed in the full text. Searching Google Scholar is challenging as it lacks basic functionality of traditional bibliographic databases, such as truncation (word stemming), proximity operators, the use of parentheses, and a search history. Additionally, search strategies are limited to a maximum of 256 characters, which means that creating a thorough search strategy can be laborious.

Whether Embase and Web of Science can be replaced by Scopus remains uncertain. We have not yet gathered enough data to be able to make a full comparison between Embase and Scopus. In 23 reviews included in this research, Scopus was searched. In 12 reviews (52%), Scopus retrieved 100% of all included references retrieved by Embase or Web of Science. In the other 48%, the recall by Scopus was suboptimal, in one occasion as low as 38%.

Of all reviews in which we searched CINAHL and PsycINFO, respectively, for 6 and 9% of the reviews, unique references were found. For CINAHL and PsycINFO, in one case each, unique relevant references were found. In both these reviews, the topic was highly related to the topic of the database. Although we did not use these special topic databases in all of our reviews, given the low number of reviews where these databases added relevant references, and observing the special topics of those reviews, we suggest that these subject databases will only add value if the topic is related to the topic of the database.

Many articles written on this topic have calculated overall recall of several reviews, instead of the effects on all individual reviews. Researchers planning a systematic review generally perform one review, and they need to estimate the probability that they may miss relevant articles in their search. When looking at the overall recall, the combination of Embase and MEDLINE and either Google Scholar or Web of Science could be regarded sufficient with 96% recall. This number however is not an answer to the question of a researcher performing a systematic review, regarding which databases should be searched. A researcher wants to be able to estimate the chances that his or her current project will miss a relevant reference. However, when looking at individual reviews, the probability of missing more than 5% of included references found through database searching is 33% when Google Scholar is used together with Embase and MEDLINE and 30% for the Web of Science, Embase, and MEDLINE combination. What is considered acceptable recall for systematic review searches is open for debate and can differ between individuals and groups. Some reviewers might accept a potential loss of 5% of relevant references; others would want to pursue 100% recall, no matter what cost. Using the results in this research, review teams can decide, based on their idea of acceptable recall and the desired probability which databases to include in their searches.

### Strengths and limitations

We did not investigate whether the loss of certain references had resulted in changes to the conclusion of the reviews. Of course, the loss of a minor non-randomized included study that follows the systematic review’s conclusions would not be as problematic as losing a major included randomized controlled trial with contradictory results. However, the wide range of scope, topic, and criteria between systematic reviews and their related review types make it very hard to answer this question.

We found that two databases previously not recommended as essential for systematic review searching, Web of Science and Google Scholar, were key to improving recall in the reviews we investigated. Because this is a novel finding, we cannot conclude whether it is due to our dataset or to a generalizable principle. It is likely that topical differences in systematic reviews may impact whether databases such as Web of Science and Google Scholar add value to the review. One explanation for our finding may be that if the research question is very specific, the topic of research might not always be mentioned in the title and/or abstract. In that case, Google Scholar might add value by searching the full text of articles. If the research question is more interdisciplinary, a broader science database such as Web of Science is likely to add value. The topics of the reviews studied here may simply have fallen into those categories, though the diversity of the included reviews may point to a more universal applicability.

Although we searched PubMed as supplied by publisher separately from MEDLINE in Ovid, we combined the included references of these databases into one measurement in our analysis. Until 2016, the most complete MEDLINE selection in Ovid still lacked the electronic publications that were already available in PubMed. These could be retrieved by searching PubMed with the subset as supplied by publisher. Since the introduction of the more complete MEDLINE collection *Epub Ahead of Print*, *In-Process & Other Non-Indexed Citations*, *and Ovid MEDLINE®*, the need to separately search PubMed as supplied by publisher has disappeared. According to our data, PubMed’s “as supplied by publisher” subset retrieved 12 unique included references, and it was the most important addition in terms of relevant references to the four major databases. It is therefore important to search MEDLINE including the “Epub Ahead of Print, In-Process, and Other Non-Indexed Citations” references.

These results may not be generalizable to other studies for other reasons. The skills and experience of the searcher are one of the most important aspects in the effectiveness of systematic review search strategies [23–25]. The searcher in the case of all 58 systematic reviews is an experienced biomedical information specialist. Though we suspect that searchers who are not information specialists or librarians would have a higher possibility of less well-constructed searches and searches with lower recall, even highly trained searchers differ in their approaches to searching. For this study, we searched to achieve as high a recall as possible, though our search strategies, like any other search strategy, still missed some relevant references because relevant terms had not been used in the search. We are not implying that a combined search of the four recommended databases will never result in relevant references being missed, rather that failure to search any one of these four databases will likely lead to relevant references being missed. Our experience in this study shows that additional efforts, such as hand searching, reference checking, and contacting key players, should be made to retrieve extra possible includes.

Based on our calculations made by looking at random systematic reviews in PubMed, we estimate that 60% of these reviews are likely to have missed more than 5% of relevant references only because of the combinations of databases that were used. That is with the generous assumption that the searches in those databases had been designed sensitively enough. Even when taking into account that many searchers consider the use of Scopus as a replacement of Embase, plus taking into account the large overlap of Scopus and Web of Science, this estimate remains similar. Also, while the Scopus and Web of Science assumptions we made might be true for coverage, they are likely very different when looking at recall, as Scopus does not allow the use of the full features of a thesaurus. We see that reviewers rarely use Web of Science and especially Google Scholar in their searches, though they retrieve a great deal of unique references in our reviews. Systematic review searchers should consider using these databases if they are available to them, and if their institution lacks availability, they should ask other institutes to cooperate on their systematic review searches.

The major strength of our paper is that it is the first large-scale study we know of to assess database performance for systematic reviews using prospectively collected data. Prior research on database importance for systematic reviews has looked primarily at whether included references could have theoretically been found in a certain database, but most have been unable to ascertain whether the researchers actually found the articles in those databases [10, 12, 16, 17, 26]. Whether a reference is available in a database is important, but whether the article can be found in a precise search with reasonable recall is not only impacted by the database’s coverage. Our experience has shown us that it is also impacted by the ability of the searcher, the accuracy of indexing of the database, and the complexity of terminology in a particular field. Because these studies based on retrospective analysis of database coverage do not account for the searchers’ abilities, the actual findings from the searches performed, and the indexing for particular articles, their conclusions lack immediate translatability into practice. This research goes beyond retrospectively assessed coverage to investigate real search performance in databases. Many of the articles reporting on previous research concluded that one database was able to retrieve most included references. Halladay et al. [10] and van Enst et al. [16] concluded that databases other than MEDLINE/PubMed did not change the outcomes of the review, while Rice et al. [17] found the added value of other databases only for newer, non-indexed references. In addition, Michaleff et al. [26] found that Cochrane CENTRAL included 95% of all RCTs included in the reviews investigated. Our conclusion that Web of Science and Google Scholar are needed for completeness has not been shared by previous research. Most of the previous studies did not include these two databases in their research.

## Conclusions

We recommend that, regardless of their topic, searches for biomedical systematic reviews should combine Embase, MEDLINE (including electronic publications ahead of print), Web of Science (Core Collection), and Google Scholar (the 200 first relevant references) at minimum. Special topics databases such as CINAHL and PsycINFO should be added if the topic of the review directly touches the primary focus of a specialized subject database, like CINAHL for focus on nursing and allied health or PsycINFO for behavioral sciences and mental health. For reviews where RCTs are the desired study design, Cochrane CENTRAL may be similarly useful. Ignoring one or more of the databases that we identified as the four key databases will result in more precise searches with a lower number of results, but the researchers should decide whether that is worth the >increased probability of losing relevant references. This study also highlights once more that searching databases alone is, nevertheless, not enough to retrieve all relevant references.

Future research should continue to investigate recall of actual searches beyond coverage of databases and should consider focusing on the most optimal database combinations, not on single databases.

## Additional files

Additional file 1: Reviews included in the research**.** References to the systematic reviews published by Erasmus MC authors that were included in the research. (DOCX 19 kb)
