# Supplementary material for: Optimal database combinations for literature searches in systematic reviews: a prospective exploratory study
Source: Syst Rev. 2017 Dec 6;6:245. doi: 10.1186/s13643-017-0644-y (PMC5718002; doi:10.1186/s13643-017-0644-y)
Supplement: Supplementary file 1 — Reviews included in the research. References to the systematic reviews published by Erasmus MC authors that were included in the research. (DOCX 19 kb) [file 13643_2017_644_MOESM1_ESM.docx]

Optimal database combinations – Bramer, Rethlefsen, Kleijnen and Franco

Reviews included in the research

Adank MC, van Dorp W, Smit M, van Casteren NJ, Laven JS, Pieters R, et al. Electroejaculation as a method of fertility preservation in boys diagnosed with cancer: a single-center experience and review of the literature. Fertil Steril. 2014;102(1):199-205 e1.

Ahmadi AR, Lafranca JA, Claessens LA, Imamdi RM, JN IJ, Betjes MG, et al. Shifting paradigms in eligibility criteria for live kidney donation: a systematic review. Kidney Int. 2015;87(1):31-45.

Atiq F, van den Bemt PM, Leebeek FW, van Gelder T, Versmissen J. A systematic review on the accumulation of prophylactic dosages of low-molecular-weight heparins (LMWHs) in patients with renal insufficiency. Eur J Clin Pharmacol. 2015;71(8):921-9.

Baas M, Duraku LS, Corten EM, Mureau MA. A systematic review on the sensory reinnervation of free flaps for tongue reconstruction: Does improved sensibility imply functional benefits? J Plast Reconstr Aesthet Surg. 2015;68(8):1025-35.

Balak DM, Fallah Arani S, Hajdarbegovic E, Hagemans CA, Bramer WM, Thio HB, et al. Efficacy, effectiveness and safety of fumaric acid esters in the treatment of psoriasis: a systematic review of randomized and observational studies. Br J Dermatol. 2016;175(2):250-62.

Bijlard E, Steltenpool S, Niessen FB. Intralesional 5-fluorouracil in keloid treatment: a systematic review. Acta Derm Venereol. 2015;95(7):778-82.

Blokker BM, Wagensveld IM, Weustink AC, Oosterhuis JW, Hunink MG. Non-invasive or minimally invasive autopsy compared to conventional autopsy of suspected natural deaths in adults: a systematic review. Eur Radiol. 2016;26(4):1159-79.

Boersema GS, Grotenhuis N, Bayon Y, Lange JF, Bastiaansen-Jenniskens YM. The Effect of Biomaterials Used for Tissue Regeneration Purposes on Polarization of Macrophages. Biores Open Access. 2016;5(1):6-14.

Braun KV, Voortman T, Dhana K, Troup J, Bramer WM, Troup J, et al. The role of DNA methylation in dyslipidaemia: A systematic review. Prog Lipid Res. 2016;64:178-91.

Caron CJ, Pluijmers BI, Joosten KF, Mathijssen IM, van der Schroeff MP, Dunaway DJ, et al. Feeding difficulties in craniofacial microsomia: a systematic review. Int J Oral Maxillofac Surg. 2015;44(6):732-7.

Caron CJ, Pluijmers BI, Joosten KF, Mathijssen IM, van der Schroeff MP, Dunaway DJ, et al. Obstructive sleep apnoea in craniofacial microsomia: a systematic review. Int J Oral Maxillofac Surg. 2015;44(5):592-8.

Claessen FM, de Vos RJ, Reijman M, Meuffels DE. Predictors of primary Achilles tendon ruptures. Sports Med. 2014;44(9):1241-59.

Cnossen MC, Scholten AC, Lingsma HF, Synnot A, Tavender E, Gantner D, et al. Adherence to Guidelines in Adult Patients with Traumatic Brain Injury: A Living Systematic Review. J Neurotrauma. 2016.

De Bruijn KM, van Eijck CH. New-onset diabetes after distal pancreatectomy: a systematic review. Ann Surg. 2015;261(5):854-61.

de Jong MH, Kamperman AM, Oorschot M, Priebe S, Bramer W, van de Sande R, et al. Interventions to Reduce Compulsory Psychiatric Admissions: A Systematic Review and Meta-analysis. JAMA Psychiatry. 2016;73(7):657-64.

de Lijster JM, Dierckx B, Utens EM, Verhulst FC, Zieldorff C, Dieleman GC, et al. The Age of Onset of Anxiety Disorders: A Meta-analysis. Can J Psychiatry. 2016.

De Lima A, Galjart B, Wisse PH, Bramer WM, van der Woude CJ. Does lower gastrointestinal endoscopy during pregnancy pose a risk for mother and child? - a systematic review. BMC Gastroenterol. 2015;15:15.

de Vos RJ, Windt J, Weir A. Strong evidence against platelet-rich plasma injections for chronic lateral epicondylar tendinopathy: a systematic review. Br J Sports Med. 2014;48(12):952-6.

de Vos-Kerkhof E, Geurts DH, Wiggers M, Moll HA, Oostenbrink R. Tools for 'safety netting' in common paediatric illnesses: a systematic review in emergency care. Arch Dis Child. 2016;101(2):131-9.

Garcia AH, Voortman T, Baena CP, Chowdhurry R, Muka T, Jaspers L, et al. Maternal weight status, diet, and supplement use as determinants of breastfeeding and complementary feeding: a systematic review and meta-analysis. Nutr Rev. 2016;74(8):490-516.

Hakim MS, Wang W, Bramer WM, Geng J, Huang F, de Man RA, et al. The global burden of hepatitis E outbreaks: a systematic review. Liver Int. 2017;37(1):19-31.

Hogendoorn W, Lavida A, Hunink MG, Moll FL, Geroulakos G, Muhs BE, et al. Open repair, endovascular repair, and conservative management of true splenic artery aneurysms. J Vasc Surg. 2014;60(6):1667-76 e1.

Hosnijeh FS, Runhaar J, van Meurs JB, Bierma-Zeinstra SM. Biomarkers for osteoarthritis: Can they be used for risk assessment? A systematic review. Maturitas. 2015;82(1):36-49.

Jaspers L, Feys F, Bramer WM, Franco OH, Leusink P, Laan ET. Efficacy and Safety of Flibanserin for the Treatment of Hypoactive Sexual Desire Disorder in Women: A Systematic Review and Meta-analysis. JAMA Intern Med. 2016;176(4):453-62.

Koning IV, Tielemans MJ, Hoebeek FE, Ecury-Goossen GM, Reiss IK, Steegers-Theunissen RP, et al. Impacts on prenatal development of the human cerebellum: a systematic review. J Matern Fetal Neonatal Med. 2016:1-7.

Kragt L, Dhamo B, Wolvius EB, Ongkosuwito EM. The impact of malocclusions on oral health-related quality of life in children-a systematic review and meta-analysis. Clin Oral Investig. 2016;20(8):1881-94.

Lafranca JA, JN IJ, Betjes MG, Dor FJ. Body mass index and outcome in renal transplant recipients: a systematic review and meta-analysis. BMC Med. 2015;13:111.

Leermakers ET, Moreira EM, Kiefte-de Jong JC, Darweesh SK, Visser T, Voortman T, et al. Effects of choline on health across the life course: a systematic review. Nutr Rev. 2015;73(8):500-22.

Li J, Hernanda PY, Bramer WM, Peppelenbosch MP, van Luijk J, Pan Q. Anti-tumor effects of metformin in animal models of hepatocellular carcinoma: a systematic review and meta-analysis. PLoS One. 2015;10(6):e0127967.

Liebregts M, Vriesendorp PA, Mahmoodi BK, Schinkel AF, Michels M, ten Berg JM. A Systematic Review and Meta-Analysis of Long-Term Outcomes After Septal Reduction Therapy in Patients With Hypertrophic Cardiomyopathy. JACC Heart Fail. 2015;3(11):896-905.

Ligthart KA, Buitendijk L, Koes BW, van Middelkoop M. The association between ethnicity, socioeconomic status and compliance to pediatric weight-management interventions - A systematic review. Obes Res Clin Pract. 2016.

Luk F, de Witte SF, Bramer WM, Baan CC, Hoogduijn MJ. Efficacy of immunotherapy with mesenchymal stem cells in man: a systematic review. Expert Rev Clin Immunol. 2015;11(5):617-36.

Mosler AB, Agricola R, Weir A, Holmich P, Crossley KM. Which factors differentiate athletes with hip/groin pain from those without? A systematic review with meta-analysis. Br J Sports Med. 2015;49(12):810.

Muka T, Koromani F, Portilla E, O'Connor A, Bramer WM, Troup J, et al. The role of epigenetic modifications in cardiovascular disease: A systematic review. Int J Cardiol. 2016;212:174-83.

Nano J, Muka T, Cepeda M, Voortman T, Dhana K, Brahimaj A, et al. Association of circulating total bilirubin with the metabolic syndrome and type 2 diabetes: A systematic review and meta-analysis of observational evidence. Diabetes Metab. 2016;42(6):389-97.

Rodenburg-Vlot MB, Ruytjens L, Oostenbrink R, Goedegebure A, van der Schroeff MP. Systematic Review: Incidence and Course of Hearing Loss Caused by Bacterial Meningitis: In Search of an Optimal Timed Audiological Follow-up. Otol Neurotol. 2016;37(1):1-8.

Roelants JA, de Jonge RC, Steegers-Theunissen RP, Reiss IK, Joosten KF, Vermeulen MJ. Prenatal markers of neonatal fat mass: A systematic review. Clin Nutr. 2016;35(5):995-1007.

Rokx C, Rijnders BJ, van Laar JA. Treatment of multicentric Castleman&rsquo;s disease in HIV-1 infected and uninfected patients: a systematic review. Neth J Med. 2015;73(5):202-10.

Schoots IG, Roobol MJ, Nieboer D, Bangma CH, Steyerberg EW, Hunink MG. Magnetic resonance imaging-targeted biopsy may enhance the diagnostic accuracy of significant prostate cancer detection compared to standard transrectal ultrasound-guided biopsy: a systematic review and meta-analysis. Eur Urol. 2015;68(3):438-50.

Serner A, van Eijck CH, Beumer BR, Holmich P, Weir A, de Vos RJ. Study quality on groin injury management remains low: a systematic review on treatment of groin pain in athletes. Br J Sports Med. 2015;49(12):813.

Simons SM, Cillessen FH, Hazelzet JA. Determinants of a successful problem list to support the implementation of the problem-oriented medical record according to recent literature. BMC Med Inform Decis Mak. 2016;16:102.

Strang SG, Van Lieshout EM, Van Waes OJ, Verhofstad MH. Prevalence and mortality of abdominal compartment syndrome in severely injured patients: A systematic review. J Trauma Acute Care Surg. 2016;81(3):585-92.

Suijkerbuijk MA, Reijman M, Lodewijks SJ, Punt J, Meuffels DE. Hamstring Tendon Regeneration After Harvesting: A Systematic Review. Am J Sports Med. 2015;43(10):2591-8.

Suker M, Beumer BR, Sadot E, Marthey L, Faris JE, Mellon EA, et al. FOLFIRINOX for locally advanced pancreatic cancer: a systematic review and patient-level meta-analysis. Lancet Oncol. 2016;17(6):801-10.

Ten Kate CA, Tibboel D, Kraemer US. B-type natriuretic peptide as a parameter for pulmonary hypertension in children. A systematic review. Eur J Pediatr. 2015;174(10):1267-75.

Tielemans MJ, Garcia AH, Peralta Santos A, Bramer WM, Luksa N, Luvizotto MJ, et al. Macronutrient composition and gestational weight gain: a systematic review. Am J Clin Nutr. 2016;103(1):83-99.

Tromp K, Zwaan CM, van de Vathorst S. Motivations of children and their parents to participate in drug research: a systematic review. Eur J Pediatr. 2016;175(5):599-612.

van der Does Y, Rood PP, Haagsma JA, Patka P, van Gorp EC, Limper M. Procalcitonin-guided therapy for the initiation of antibiotics in the ED: a systematic review. Am J Emerg Med. 2016;34(7):1286-93.

van der Valk JP, Dubois AE, Gerth van Wijk R, Wichers HJ, de Jong NW. Systematic review on cashew nut allergy. Allergy. 2014;69(6):692-8.

van Dijk GM, Maneva M, Colpani V, Dhana K, Muka T, Jaspers L, et al. The association between vasomotor symptoms and metabolic health in peri- and postmenopausal women: a systematic review. Maturitas. 2015;80(2):140-7.

van Mol MM, Kompanje EJ, Benoit DD, Bakker J, Nijkamp MD. The Prevalence of Compassion Fatigue and Burnout among Healthcare Professionals in Intensive Care Units: A Systematic Review. PLoS One. 2015;10(8):e0136955.

van Waardhuizen CN, Langhout M, Ly F, Braun L, Genders TS, Petersen SE, et al. Diagnostic performance and comparative cost-effectiveness of non-invasive imaging tests in patients presenting with chronic stable chest pain with suspected coronary artery disease: a systematic overview. Curr Cardiol Rep. 2014;16(10):537.

Vargas KG, Milic J, Zaciragic A, Wen KX, Jaspers L, Nano J, et al. The functions of estrogen receptor beta in the female brain: A systematic review. Maturitas. 2016;93:41-57.

Voogt L, de Vries J, Meeus M, Struyf F, Meuffels D, Nijs J. Analgesic effects of manual therapy in patients with musculoskeletal pain: a systematic review. Man Ther. 2015;20(2):250-6.

Wesseloo R, Kamperman AM, Munk-Olsen T, Pop VJ, Kushner SA, Bergink V. Risk of Postpartum Relapse in Bipolar Disorder and Postpartum Psychosis: A Systematic Review and Meta-Analysis. Am J Psychiatry. 2016;173(2):117-27.

Wu Z, van de Haar RC, Sparreboom CL, Boersema GS, Li Z, Ji J, et al. Is the intraoperative air leak test effective in the prevention of colorectal anastomotic leakage? A systematic review and meta-analysis. Int J Colorectal Dis. 2016;31(8):1409-17.

Xavier Harmeling J, Kouwenberg CA, Bijlard E, Burger KN, Jager A, Mureau MA. The effect of immediate breast reconstruction on the timing of adjuvant chemotherapy: a systematic review. Breast Cancer Res Treat. 2015;153(2):241-51.

Younge JO, Gotink RA, Baena CP, Roos-Hesselink JW, Hunink MG. Mind-body practices for patients with cardiac disease: a systematic review and meta-analysis. Eur J Prev Cardiol. 2015;22(11):1385-98.
